# The Forensic High and Intensive Care Monitor: Measurement Properties of a Model Fidelity Scale for Contact-Based Care in Forensic Psychiatry

**DOI:** 10.1007/s10488-021-01185-9

**Published:** 2022-02-16

**Authors:** Sylvia Gerritsen, Guy A. M. Widdershoven, Anne L. van Melle, Henrica C. W. de Vet, Yolande Voskes

**Affiliations:** 1 Department of Ethics, Law and Humanities, Amsterdam UMC, VU University Amsterdam, Amsterdam, The Netherlands; 2 GGZ inGeest, Amsterdam, The Netherlands; 3 Department of Epidemiology and Data Science, Amsterdam UMC, VU University Medical Center, Amsterdam, The Netherlands; 4 GGz Breburg, Tilburg, The Netherlands; 5 Tranzo Scientific Center for Care and Wellbeing, Tilburg School of Social and Behavioral Sciences, Tilburg University, Tilburg, The Netherlands

**Keywords:** Forensic mental healthcare, Forensic High and Intensive Care (FHIC), Psychometric properties, Model fidelity scale, Audits

## Abstract

Forensic High and Intensive Care (FHIC) has recently been developed as a new care model in Dutch forensic psychiatry. FHIC aims to provide contact-based care. To support Dutch forensic care institutions in the implementation of the model, a model fidelity scale was developed called the FHIC monitor. The aim of this study was to assess the inter-rater reliability, content validity, and construct validity of the FHIC monitor. A multi-methods design was used, combining qualitative and quantitative research. To collect data, audits and focus group meetings were organized to score care at individual wards with the monitor and get feedback from auditors and audit receiving teams about the quality of the monitor. In total, fifteen forensic mental healthcare institutions participated. The instrument showed acceptable inter-rater reliability and content validity, and a significant difference between expected high and low scoring institutions, supporting construct validity. The instrument can be used as a valid instrument to measure the level of implementation of the FHIC model on forensic psychiatric wards in the Netherlands.

**Supplementary Information:**

The online version contains supplementary material available at 10.1007/s10488-021-01185-9.

## Introduction

In recent years, the reduction of coercive measures in forensic mental healthcare, especially concerning the use of seclusion, has received increasing attention (Laiho et al., 2016). Seclusion is known to have a negative impact on patients, care professionals and care institutions (Goulet et al., 2017; Haw et al., 2011; Keski-Valkama et al., 2010). Goulet, et al (2017) concluded that there is international consensus that seclusion should be reduced in (forensic) mental health care. However, a reduction of seclusion in forensic psychiatry is complicated by the explicit focus on safety (Goulet, et al., 2017). Care professionals in forensic psychiatry tend to focus on control for ensuring safety, especially in challenging situations such as a crisis. Instead of control-based care, contact-based care is suggested to contribute to less aggression, prevention of care disruption, and reduction of coercive measures as seclusion (Ros et al., 2013; van der Helm et al., 2011).

To provide contact-based care in crisis situations, a new care model was developed in Dutch forensic psychiatry, called Forensic High and Intensive Care (FHIC). The FHIC model is based on evidence and best-practices (so, a combination of evidence- and practice-based) from both forensic- and regular psychiatry. The development process consisted of six expert meetings with all important stakeholders from forensic mental health care and regular mental health care. Psychiatrist, nurses, psychologists, peer providers, managers and policy makers were present at these meetings. During these meetings the participants reflected on the basis principles of the already existing HIC model for regular psychiatry (Voskes et al., 2021), like stepped care, the reduction of coercive measures and contact-based care. These elements are also relevant to forensic psychiatry. Yet, the FHIC model also contains elements that are specifically relevant to forensic psychiatry. These are risk assessment, response to and evaluation after incidents, and a team composition that is consistent with forensic care (Bogaerts et al., 2018; Van de Sande et al., 2011). Furthermore, the FHIC model includes the theory of limit setting and the principles of the open institutional climate, focused on support, growth, a positive atmosphere and a reduction of repression (Maguire et al., 2014; Ros et al., 2013). At the moment, the FHIC model is being implemented by Dutch forensic care institutions. As the FHIC model is a multifaceted care intervention in a complex field, it is necessary to support care professionals in this process. Implementation of a complex intervention requires specific attention (Ewington, 2016). For a successful and sustainable implementation, professionals are in need of strategies and tools to constantly foster awareness (Mann-Poll et al., 2018).

To measure the degree of implementation and support care professionals in the implementation process, a model fidelity scale is useful, as it identifies differences in practices between mental healthcare institutions (Bond et al., 2011; van Melle et al., 2019; van Weeghel, 2020). Therefore, the FHIC monitor was developed, based on the FHIC model, expert consensus and scientific research. The FHIC monitor is inspired by the High and Intensive Care (HIC) monitor (van Melle et al., 2019).

In order to secure assessment of implementation of FHIC, the FHIC monitor needs to be valid and reliable for use in forensic psychiatry. Therefore, a careful assessment of the psychometric properties of the FHIC monitor is needed (Mokkink et al., 2010). This study aims to assess the interrater reliability, the content validity, and the construct validity of the FHIC monitor.

## METHODS

### Instrument

The FHIC monitor was developed in two steps. First, the precursor HIC monitor was used as a basis for formulating items, specifying them for the forensic setting and adjusting them based on the FHIC model. Second, after a try-out in eight audits, the monitor was evaluated, based on analysis of the results of the audits, feedback of the auditors and the audit receiving teams. It appeared that the monitor still insufficiently reflected forensic practice, for instance because the distinction between various levels of security was lacking. In Dutch forensic psychiatry, there are different types of clinic settings where patients remain, ranging from a low, medium to high level of security. Consequently, the monitor was adjusted textually and in terms of content, making a distinction between low and high security levels, making requirements for team composition more in line with forensic psychiatry, emphasizing consultation of referring care professionals, and including internal referrals, which specifically applies to high security settings. Some items were removed, for example: "laws and regulation" (which is evident in forensic psychiatry). A new item was added, focusing on care for patients at the ward after an incident. These changes made the FHIC monitor further in line with the FHIC model and the forensic practice. The adjusted monitor was presented at a national meeting of auditors and representatives of care institutions and accepted by all participants. This new version of the monitor served as a basis for the validation process.

### Design

For this study, a multi-methods design was used, combining qualitative and quantitative research.

### Participants

Dutch forensic mental healthcare institutions which had started with the implementation of FHIC or had the intention to do so, were invited to participate in this study. They were approached by the researchers and the Dutch Expertise Center on Forensic Psychiatry. In total, fifteen institutions participated, and settings with a low security (n = 6), medium security (n = 2) and high security (= 7) level were included. Within these care institutions, data was collected at one of the wards. Information was gathered on the level of the ward, care professionals and patients.

### Data Collection

Data collection took place by audits and focus groups in the period between 2018 and 2019.

#### Audits

At each participating care institution, data was collected by means of an audit of two care professionals who individually scored the items of the monitor for one ward. The initial plan was to perform the audits with three auditors: two care professionals and a peer provider. Due to the shortage of peer providers in forensic psychiatry, it was not possible to accomplish this for each audit. Therefore, only the scores of the care professionals have been used as data. The auditors received a one-day training by the researchers and the FHIC project coordinator of the Dutch Expertise Center on Forensic Psychiatry. An experienced HIC auditor gave advice based on experience with the auditing process during the first training. In subsequent training days for new auditors, this role was taken by experienced FHIC auditors. During the data collection period, auditors and researchers shared their experiences in regular meetings. In this way, a Community of Practice (CoP) of FHIC care professionals was established.

During an audit, a participating ward was scored during a site visit by two auditors of two other care institutions. The program of the site visit contained the following activities: a tour on the ward, interviews (with a patient and care professionals with various disciplinary background), joining the daily multidisciplinary meeting and performing a patient file check (to check the documentation of certain elements of the monitor). Furthermore, auditors received information on the number of beds and staff, and the mean duration of admission. After the audit, the auditors independently returned a form with scores on the items of the monitor to the researchers. On this form, they included a short argumentation per item. In addition, they wrote a brief general impression, and identified strong points and suggestions for improvements. Based on the auditors’ forms the researchers made a preliminary report per ward.

#### Focus Groups

Some weeks after the audit, the researchers visited the audit-receiving ward to discuss the obtained scores in a focus group meeting. Care professionals from the ward with various disciplinary backgrounds and work experiences participated. The scores of the auditors were compared with the expectations of the team per item. Also, participants were asked to comment on the relevance, comprehensibility and completeness of FHIC monitor. The mean duration of the focus group meetings was 2 h. The researchers made notes of the meeting, and when permission was obtained from the participants audio recordings were made.

### Data Analysis

To analyse the degree of validity and reliability of the FHIC monitor, we studied three measurement properties of the FHIC monitor. For the quantitative analysis, SPSS (Statistical Package for the Social Sciences) version 20 were used. Below is an explanation per analysis:

#### Inter-Rater Reliability

The inter-rater reliability was studied to assess to what extent measurements led to the same result. Therefore, the independent scores of the two auditors were compared per audit, expressed in a percentage of agreement (de Vet et al., 2006). In this, an agreement with one point difference was allowed in case of an item with 5 scoring options. For items with only 2, 3 or 4 scoring options, only the exact agreement was examined. A percentage of at least 75% agreement was considered as an acceptable inter-rater reliability (Chaturvedi & Shweta, 2015; Stemler, 2004). This analysis is comparable to the study designed to validate the HIC monitor (van Melle et al., 2019).

#### Content Validity

The content validity, i.e. the degree of relevance, comprehensibility and completeness of each domain and item of the instrument (de Vet et al., 2011). We evaluated the content validity of the FHIC monitor by a qualitative analysis of the feedback from the focus groups with audit-receiving teams and meetings with auditors. In meetings with the auditors, items were reflected upon that had emerged from the focus groups with audit receiving teams related to content validity. Auditors could also bring in other items to discuss. Furthermore, for each item the mean and standard deviation was calculated. Structurally high or low scoring items were examined to see whether they were appropriately formulated (Mokkink et al., 2010). Furthermore, the results of the content validity were compared to the results of the inter-rater reliability. Items which did not sufficiently meet both measurement properties were further analysed and discussed by the researchers based on the qualitative data.

#### Construct Validity

As there is no golden standard, we evaluated the construct validity of the FHIC by hypotheses-testing. By lack of comparative instruments, we used known group validity to assess to what extent the FHIC monitor is able to identify expected differences between groups (Mokkink et al., 2010). We formulated a hypothesis predicting an expected higher and lower scoring group. The determination of the expected higher and lower scoring groups, was based on the duration of the implementation process and active involvement in the FHIC project per participating ward. It is our assumption and experience that institutions that have been in the implementation process longer will be further along in the implementation process. While some of the participating institutions had already actively started implementing the FHIC model, others had yet to begin. To strengthen the analyses we also formulated three sub hypotheses, referring to items reflecting attitude, FHIC work routine, and evaluation of coercive measures. Because these three topics represent the core elements of the FHIC model, we expected that wards who had been actively working on the implementation of FHIC would show a higher score on the corresponding items. For the analysis, the difference between both groups was identified using a t-test with significance level p < 0.05. A sample size calculation based on previously described HIC research showed that with the same spread between the two groups, a number of seven wards per group can identify a statistically significant difference (with SD = 0.33, p < 0.05). A minimum of fourteen participating institutions was therefore considered sufficient.

## Results

**Fig. 1:**
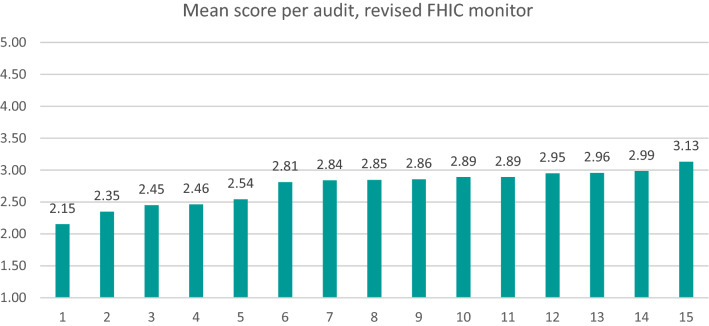
An overview of the mean scores per audit (n = 15), performed on the basis of the revised FHIC monitor

Fifteen audits were conducted based on the monitor. Figure 1 shows an overview of the mean scores per audit. Below the results for each measurement characteristic are described.

### Inter-rater Reliability

Table 1 provides an overview of the inter-rater reliability per item. For each item, the average score, standard deviation and percentages of agreement between auditors are shown. In total, 42 of the 69 (sub)items met the 75% agreement criterion and therefore show an acceptable inter-rater reliability. Of those items, some expressed a high percentage of agreement and a number of items showed a low inter-rater reliability. For these items, possible reasons for these differences were identified. Most often, the formulation of the items appeared to be unclear or not specific enough. These items were reconsidered in the process of finalizing the monitor (see below).

**Table 1:** An overview of the mean score (SD), percentage exact agreement and percentage if 1-point difference is allowed per item on the revised FHIC monitor

Item		Average score (SD)	% Exact agreement	% Agreement if 1- point difference is allowed
Team structure				
1a*	Small ST caseload: day	2.60 (1,62)	73.33	
1b*	Small ST caseload: evening	2.60 (1.62)	73.33	
1c*	Small ST caseload: night	2.08 (1.39)	66.67	
2	Stepped care	3.30 (1.58)	40.00	60.00
3	Nurse/social worker	2.68 (1.63)	86.67	100.00
4	Psychiatrists	2.48 (1.47)	40.00	80.00
5a*	(Coordinating) practitioner: disciplines	2.98 (1.42)	66.67	
5b	(Coordinating) practitioner: FTE	2.83 (1.50)	46.67	66.67
6	(Family) peer providers	1.13 (0.51)	86.67	93.33
7*	Activity supervisors	3.33 (1.15)	53.33	
8	Supervisors/team leaders	2.63 (1.49)	60.00	80.00
9	Extra disciplines	4.18 (0.54)	86.67	100.00
10a	Team relationship: experience	4.03 (1.17)	46.67	60.00
10b	Team relationship: blended	3.80 (0.84)	60.00	80.00
11	Staffing	3.38 (1.20)	33.33	93.33
Team processes				
12	Vision	1.95 (1.02)	60.00	86.67
13	Hospitality	3.33 (1.27)	33.33	73.33
14*	Presence	2.55 (1.38)	60.00	
15	Attitude/treatment	2.28 (1.18)	53.33	80.00
16*	Prevention repression	1.75 (1.07)	60.00	
17	Care alignment meeting (ZAG)	2.03 (1.47)	66.67	93.33
18	Treatment plan	2.55 (1.79)	60.00	66.67
19	Digital whiteboard	2.08 (1.35)	53.33	86.67
20	Duration of the stay at FHIC	1.55 (1.16)	93.33	93.33
21a*	ICU care process and consultation	1.65 (1.22)	80.00	
21b*	ESR care process and consultation	2.50 (1.38)	40.00	
Diagnostics, treatment and treatment interventions				
22*	Guidelines	3.05 (1.64)	53.33	
23*	Initial diagnostics	3.15 (1.64)	60.00	
24a	General examination: history	2.43 (1.60)	40.00	60.00
24b	General examination: medical	3.78 (1.47)	53.33	73.33
25a*	Risk assessment: short term	2.25 (1.32)	80.00	
25b	Risk assessment: long term	4.40 (1.26)	73.33	80.00
26*	Conflict management and personal security	4.30 (1.14)	80.00	
27a	Medication policy	4.50 (0.87)	60.00	86.67
27b*	Early and acute intervention medication	3.05 (1.52)	40.00	
28	Addiction care	2.58 (1.39)	20.00	80.00
29	Structural information	4.23 (1.19)	53.33	80.00
30	Day activities	2.95 (1.20)	73.33	93.33
31	Family interventions	2.73 (1.05)	40.00	93.33
Organisation of care				
32*	Admission and discharge	2.30 (1.65)	66.67	
33	Waiting list	2.73 (1.92)	53.33	66.67
34a	Transition: admission	1.40 (0,97)	80.00	86.67
34b	Transition: admission/discharge	1.73 (1.12)	53.33	80.00
Monitoring				
35	Use ROM (Routine Outcome Monitoring)	2.63 (0.99)	66.67	93.33
36a	FHIC improvement curve	2.08 (1.44)	53.33	80.00
36b	Work environment	2.23 (1.46)	73.33	80.00
36c	Institutional environment	1.63 (1.18)	73.33	93.33
Professionalization				
37*	Reflection on own actions	3.10 (1.41)	73.33	
38	Education	2.48 (0.67)	60.00	93.33
39*	Knowledge of integrated care	2.55 (1.52)	73.33	
40	Team spirit	3.50 (1.16)	46.67	86.67
41*	Leadership	3.65 (1.57)	66.67	
Healing environment				
42	Healing environment: HE	1.93 (1.01)	60.00	80.00
43a*	HC: individual rooms and bathrooms	3.50 (1.94)	80.00	
43b*	HC: comfort room	1.80 (1.60)	86.67	
43c*	HC: diversity in meeting rooms	3.30 (1.98)	73.33	
43d*	HC: outdoor area	4.80 (0.87)	86.67	
43e*	HC: family room	2.30 (1.87)	80.00	
43f*	HC: open desk	1.20 (0.87)	100.00	
44	The IC unit (Intensive Care)	1.73 (0.97)	46.67	80.00
45	ICU room (Intensive Care Unit)	1.75 (1.20)	53.33	80.00
46	The Extra Secure Room (ESR)/seclusion	1.88 (1.21)	60.00	80.00
Incident follow-up				
47a	Incident response: team	4.65 (0.61)	53.33	80.00
47b	Incident response: patients	3.30 (1.08)	33.33	80.00
48a	Incident evaluation: team	3.40 (1.09)	46.67	66.67
48b	Incident evaluation: patient	2.95 (1.28)	20.00	73.33
Evaluation coercive measures				
49a	Coercive measures evaluation: team	2.63 (1.51)	66.67	86.67
49b	Coercive measures evaluation: patient	2.60 (1.34)	26.67	73.33
50	Feedback on coercive measures	2.08 (1.47)	73.33	80.00

^*^ Items which only allow two or three response options: the scores 1 and 5, or 1, 3 and 5, respectively

### Content Validity

In the qualitative analysis of the focus group meetings, most items of the FHIC monitor showed a high degree of relevance, comprehensibility and completeness. Yet, some items were perceived as incomplete or not fully comprehensible by the audit-receiving wards and/or the auditors. Further analysis of the content validity was done by assessing of consistently high or low scoring items. Explanations for the high and low scoring items were sought, for instance priority or lack of priority of the items in the implementation process, or a low or high standard, resulting in a majority of high, respectively low scores. The analysis of the perceived relevance, comprehensibility and completeness, as well as the analysis of consistently high or low scoring items, provided arguments for some final adaptations (see below).

### Construct Validity

Data showed a confirmation of the hypothesis regarding the construct validity. Wards that were expected to score higher (by implementing the FHIC model longer or more actively) actually scored higher on the FHIC monitor. The data appeared to be normally distributed on both the Kolmogorov–Smirnov and Shapiro–Wilk tests. The difference in average score for the expected high scoring group (M = 2.96; SD = 0.11) and the expected low scoring group (M = 2.60; SD = 0.29) was significant (t(9.275) = 3.270, p = 0.009. The calculation for the average score on the sub hypotheses about “attitude” and “FHIC working routine” also showed significant differences between the expected high scoring group and low scoring group. However, for “evaluation of coercive measures” the expected low scoring group scored higher instead of lower than the expected high scoring group, although the difference was not significant (Table 2).

**Table 2:** Calculation for the average score of the sub hypotheses (MD (SD))

Sub hypotheses	MD expected high scoring group (SD)	MD expected low scoring group (SD)	p value
Attitude	2.80 (0.54)	1.91 (0.44)	0.005
FHIC working routine	2.56 (0.55)	1.60 (0.42)	0.002
Evaluation of coercive measures	2.43 (0.90)	2.56 (1.02)	0.792

### Finalizing the FHIC Monitor

Based on the assessment of the inter-rater reliability and the content validity, some final changes were made. These consisted of small textual changes in individual items, in order to make them more clearer. Also, several scoring options were adapted, by further specifying criteria, and changing scores with three options into a five options scale. Finally the monitor was made more user friendly by moving some items to a more adequate domain and by creating an overarching structure for the domains, distinguishing between three categories: 1) patient; 2) team; and 3) institution. The items are therefore only grouped under one of the three overarching categories. These changes implied no change of individual items. The final version was discussed and accepted in a national meeting with auditors and other representatives (n = 18) of participating care institutions. The final version of the FHIC monitor can be found in supplementary Appendix 1.

## Discussion

This study assessed the validity and reliability of the FHIC monitor, a model fidelity scale of a new care model for forensic psychiatry. The FHIC monitor reflects the core components of the FHIC model, including the principles of the treatment and care for the patient, the structure and culture of the team, and the policy regarding implementation, quality and cooperation in the institution. The results of the study show that the FHIC monitor has reasonable measurement properties. For the inter-rater reliability and content validity, most items showed acceptable outcomes. For clarification purposes, some modifications were made at item level by making changes in formulation and scoring options. Also, user friendliness was optimized by placing some items in another domain and positioning the domains in three overarching categories: 1) patient; 2) team; and 3) institution. By using hypothesis-testing of differences between groups, we were able to assess the construct validity. Despite the small group size we found a significant and considerable difference between the average score of both groups, and for the sub hypotheses about attitude and the FHIC working routines. Unexpectedly we found that expected low score groups scored slightly higher on the evaluation of coercive measures. However, we anticipated a higher score from the expected high-scoring group. This might indicate that attitude and working routines are more distinctive for the FHIC model than evaluation of coercive measures. Yet, as reduction of coercion is an important goal of FHIC, and evaluation can be regarded as contributive to fostering reduction of coercion, further research on the role of evaluation of coercive measures in FHIC practice is needed.

According to Bond and Drake, assessment of a model fidelity scale should include the following psychometric properties: “content validity, reliability, sensitivity to change, discriminative validity, adequacy of the calibration, predictive validity, and acceptability to users” (Bond & Drake, 2020, p. 879). We explicitly investigated content validity and inter-rater reliability. Issues concerning the acceptability and the scoring options were addressed in the focus group meetings and during follow-up meetings with auditors. Users experienced the FHIC monitor assessment as an intensive process, nevertheless they valued the completeness. The length of the scale is, with 50 items, higher than the recommended range between 15 and 25 items (Bond & Drake, 2020). However, it can be argued that the optimal number of items depends on the complexity and the goal for the fidelity assessment. To measure compliance with a care model, it is valuable to be complete and to make sure that all relevant topics are covered. Because completeness is important, factor analysis aimed at reducing the number of items is less appropriate.

## Strengths and Limitations

A strength of this study is the involvement of a large number of care professionals and institutions in the assessment of the FHIC monitor. As trained, external care professionals assessed the FHIC monitor, possible bias from self-assessment was prevented. Site visits were organized which is regarded as a golden standard for fidelity measurement (Becker et al., 2015; Bond & Drake, 2020). A further strength is the attention for the experienced quality of the monitor, by taking into account the feedback of auditors and teams receiving audits. This resulted in a substantial change of the monitor during the development process, and in some final adaptations as a result of the study. The need for specifying the monitor to fit the setting confirms the importance of adjusting an instrument to and in its setting, especially in forensic psychiatry (Sanchez-Balcells et al., 2018). The experiences in the development process underline the recommendation by Bond and Drake (2020) to perform a pilot because the development of a scale is a trial-and-error process. A weakness is that we did not test the final version of the monitor. However, since the changes were relatively small, we expect that our results also hold for the final monitor. A further weakness is that we were unable to use the scores of the peer providers as auditors, since they were not enough represented in the audit pool.

## Recommendations

Further research is needed to assess the sensitivity to change of the monitor. Therefore we recommend to study whether repeated audits in time result in different scores. Future research also may show whether the FHIC model will result in expected clinical outcomes and provide further indications for the FHIC model being evidence-based. We specifically recommend to investigate whether the instrument’s outcomes are related to an improvement of intended outcomes as (experienced) safety and the reduction of coercive measures.

## Conclusion

This study assessed the construct validity and inter rater reliability of the FHIC monitor, a model fidelity scale aimed to measure implementation of contact-based care in forensic psychiatry. Acceptable measurement properties were found for inter-rater reliability, construct, and content validity. Some minor textual and structural changes were done, resulting in the final version of the instrument. We propose to use the instrument to support the FHIC implementation process and meanwhile contribute to further validation, with the ultimate goal to investigate whether implementation of FHIC leads to a reduction of coercion and an improvement of quality of care.

## Supplementary Information

Below is the link to the electronic supplementary material.

Supplementary file1 (PDF 938 KB)

## Data Availability

Data supporting the findings of our study can be found in Table 2. The final FHIC monitor is available in Dutch at www.fhic.nl, an English translation can be found in supplementary Appendix 1. We encourage the reader to contact the corresponding author in case of any questions.
