# Supplementary material for: The Forensic High and Intensive Care Monitor: Measurement Properties of a Model Fidelity Scale for Contact-Based Care in Forensic Psychiatry
Source: Adm Policy Ment Health. 2022 Feb 16;49(4):587–95. doi: 10.1007/s10488-021-01185-9 (PMC9233636; doi:10.1007/s10488-021-01185-9)
Supplement: Supplementary file 1 — Supplementary file1 (PDF 938 KB) [file 10488_2021_1185_MOESM1_ESM.pdf]

FHIC Monitor

Model fidelity scale for Forensic High & Intensive Care

Final validated version

This FHIC Monitor was created in September 2019 and translated to English in 2021

The FHIC Monitor is the intellectual property of the Expertisecentrum Forensische Psychiatrie (Expertise Centre for Forensic Psychiatry)

For more information about the application of the FHIC monitor, please send an email to info@fhic.nl, FHIC@vumc.nl or visit www.fhic.nl

Amsterdam UMC
Universitair Medische Centra

Overview of the different items by domain and level

Level: patient	Level: team	Level: institution
**Domain 1. Care process and alignment**	**Domain 5. Team structure**	**Domain 8. Care organisation**
**Item 1.** Initial diagnostics<br>**Item 2.** Treatment plan<br>**Item 3.** Crisis factors<br>**Item 4.** Care alignment meeting (ZAG)<br>**Item 5.** Structural information<br>**Item 6.** Multidisciplinary consultation	**Item 19.** Small ST caseload: day (a), evening (b), night (c)<br>**Item 20.** Nurse/social worker (SPH)<br>**Item 21.** (Coordinating) practitioner: psychiatrist (a), disciplines (b), FTE (c)<br>**Item 22.** (Family) peer provider<br>**Item 23.** Activity supervisor<br>**Item 24.** Additional disciplines<br>**Item 25.** Team relationship: experience (a), mixed (b)<br>**Item 26.** Staffing	**Item 39.** Admission and discharge<br>**Item 40.** Waiting list<br>**Item 41.** Duration of the stay at FHIC<br>**Item 42.** Knowledge of integrated care<br>**Item 43.** Transition: consultation before (a) during/after admission (b)
**Domain 2. Treatment**	**Domain 6. Culture/work method**	**Domain 9. Spatial design**
**Item 7.** Somatics and lifestyle<br>**Item 8.** Medication policy (a), Early and acute intervention medication (b)<br>**Item 9.** Addiction care<br>**Item 10.** Day activities<br>**Item 11.** Use ROM (Routine Outcome Monitoring)	**Item 27.** Vision<br>**Item 28.** Hospitality<br>**Item 29.** Presence<br>**Item 30.** Attitude/treatment<br>**Item 31.** Prevention repression<br>**Item 32.** Family interventions	**Item 44.** Healing environment<br>**Item 45.** Ward: lockable rooms (a), diversity in meeting rooms (b), open desk (c), outdoor area (d), comfort room (e), family room (f)<br>**Item 46.** The IC unit (Intensive Care)<br>**Item 47.** ICU room (Intensive Care Unit)<br>**Item 48.** The Extra Secure Room (ESR)/seclusion
**Domain 3. (Prevention) coercive measures**	**Domain 7. Team development and dynamics**	**Domain 10. Quality**
**Item 12.** Stepped care<br>**Item 13.** ICU care process and consultation (a)/ESR (b)<br>**Item 14.** Coercive measures evaluation: team (a)/patient(b)<br>**Item 15.** Feedback on coercive measures	**Item 33.** Reflection on own actions<br>**Item 34.** Education & development: individual (a), team (b)<br>**Item 35.** Conflict management and personal security<br>**Item 36.** Team spirit<br>**Item 37.** Leadership<br>**Item 38.** Collaborating leader	**Item 49.** Innovation and improvement<br>**Item 50.** Quality improvement: work (a) and environment (b)
**Domain 4. Safe care**		
**Item 16.** Incident response: team (a), patients (b)<br>**Item 17.** Incident evaluation: team (a), patients (b)<br>**Item 18.** Risk assessment: short (a)/long (b) term		

Domain 1. Care process and alignment					
**Criterium**	**Score 1**	**Score 2**	**Score 3**	**Score 4**	**Score 5**
1. Initial diagnostics<br>The following initial diagnostic criteria are met within eight hours of admission:<br>- The (coordinating) practitioner applies the policy and necessary interventions until the first ZAG<br>- An initial (working) diagnosis is determined<br>- The (coordinating) practitioner communicates the policy and necessary interventions with the team<br>- Initial diagnostics are performed for both internal and external admissions	None of the criteria set are met, or the first diagnostics are not performed within the first eight hours of admission.	One of the set criteria is met	Two of the set criteria are met	Three of the set criteria are met	All four of the set criteria are met
2. Treatment plan<br>Within three working days after admission, the specific objectives for the FHIC, the necessary interventions, policies, and the working diagnosis are described in a (preliminary) treatment plan. The objectives have been established in collaboration with the patient and the referring party. In case of an internal admission, the specific objectives for the FHIC should be added to the existing treatment plan. This treatment plan can be viewed by the patient after this is discussed with the coordinating practitioner.	None of the patients has a treatment plan within three working days after admission	Less than 25% of patients have a treatment plan and the possibility of access within three working days after admission	25%-49% of patients have a treatment plan and the possibility of access within three working days after admission	50%-74% of patients have a treatment plan and the possibility of access within three working days after admission	75%-100% of patients have a treatment plan and the possibility of access within three working days after admission
3. Crisis factors<br>The main protective and triggering factors for a crisis are mapped within seven days of admission with information from the referring party and/or next of kin.	In none of the patients, this criterium is met.	This criterium is met in less than 25% of patients.	This criterium is met in 25%-49% of patients.	This criterium is met in 50%-74% of patients.	This criterium is met in 75%-100% of patients.
4. Care alignment meeting (ZAG):	None of the set criteria are met	One of the set criteria is met	Two of the set criteria are met	Three of the set criteria are met	All four of the set criteria are met

Care alignment meetings are held regularly. These are linked to the treatment plan evaluation. The treatment objectives are recorded and/or adjusted in the ZAG. The ZAG meets the following criteria:<br>- At least the following parties are present: the patient, the (coordinating) practitioner of the FHIC, and the (coordinating) practitioner of the referring party. At the patient's invitation, multiple disciplines and/or persons involved can participate.<br>- The first ZAG takes place as soon as possible after admission with the parties directly involved, at least within three working days after admission. During this meeting, agreements are made about the intended duration of the treatment.<br>- The second ZAG takes place after 3, 5 or 7 weeks (depending on security level; FPA=3, FPK=5, FPC/PPC=7 weeks).<br>- Upon termination of care, a ZAG takes place with the party taking over the care (internal or external).					
<u>5. Structural information</u><br>The FHIC provides patient information in a structural manner. This is reflected in the following criteria:<br>- Upon admission, information is provided about rights and obligations, clinical picture, treatment and related liberties policies.<br>- Information is regularly offered by the multidisciplinary team.	None of the set criteria are met	One of the set criteria is met	Two of the set criteria are met	Three of the set criteria are met	All four of the set criteria are met

- Information material (e.g., folders/patient computer) is available at the ward.<br>- Customised information is provided that takes into account the individual patient.					
<u>6. Multidisciplinary consultation (MDC)</u><br>The following criteria are met:<br>- The multidisciplinary consultation (MDC) follows a fixed structure.<br>- In principle, all team members working that day are present for the daily MDC.<br>- Team member responsibilities are made clear during the consultation.<br>- The results of the MDC are actively updated on a whiteboard/digital board.<br>- The multidisciplinary consultation always takes place by means of a digital board, is operational in the consultation room and contains relevant data.<br>- The digital board is linked to the EPF.	None or just one of the set criteria are/is met	Two or three of the set criteria are met	Four of the set criteria are met	Five of the set criteria are met	All six of the set criteria are met

Domain 2. Treatment					
**Criterium**	**Score 1**	**Score 2**	**Score 3**	**Score 4**	**Score 5**
<u>7. Somatics and lifestyle</u><br>The following criteria are met:<br>- Sufficient attention is paid to the somatic health and lifestyle of patients<br>- The team has appointed a special-task officer for somatic health<br>- Non-nursing personnel are trained to identify common somatic problems<br>- General somatic screening can be carried out within 24 hours at all times if indicated. This testing should be carried out by a general practitioner or a somatic nursing specialist.	None of the set criteria are met	One of the set criteria is met	Two of the set criteria are met	Three of the set criteria are met	All four of the set criteria are met
<u>8a. Medication policy</u><br>The following criteria are met:<br>- There is a protocol-based medication policy that is based on the most current insights/guidelines.<br>- The choice for medication has been made in consultation with the patient.<br>- The choice of medication is explained to the patient and can be repeated.<br>- The effects and side-effects of medication are checked continuously (monitoring).<br>- Structural explanations are provided on the effects and side-effects of medication by all disciplines.	None or just one of the set criteria are/is met	Two of the set criteria are met	Three of the set criteria are met	Four of the set criteria are met	All five of the set criteria are met
<u>8b. Early and acute intervention medication</u><br>The following criteria are met:	None of the set criteria are met	One of the set criteria is met	Two of the set criteria are met	Three of the set criteria are met	All four of the set criteria are met

- A protocol-based policy on the administration of early and acute intervention medication is followed.<br>- The policy is based on the most current insights/guidelines.<br>- The use of early and acute intervention medication is discussed with the patient afterwards (debriefing).<br>- The policy regarding the use of early and acute intervention medication (e.g., certain patient preferences) is adjusted as a result of the debriefing.					
<u>9. Addiction care</u><br>The following criteria are met:<br>- Addiction treatment is included in the treatment plan (diagnosis, interventions, goals).<br>- Addiction treatment is integrated into the care. There is a combined focus on (the interactions between) psychiatric and addiction problems according to 'Integrated Dual Disorder Treatment' (IDDT).<br>- Multidisciplinary guidelines (e.g., MDG for opiate addiction, MDG for alcohol addiction) are used.<br>- The wards use clear agreements about permitted and prohibited alcohol and drug use (including the consequences); this is communicated to all patients.	None of the set criteria are met	One of the set criteria is met	Two of the set criteria are met	Three of the set criteria are met	All four of the set criteria are met
<u>10. Day-time activities</u><br>All patients at the FHIC can participate in an activity programme during the day (at all times), in the evenings and on weekends	There is no patient activity programme at the FHIC.	During the day on weekdays, patients can participate in an activity programme for part of the day.	During the day on weekdays, patients can participate in an activity programme throughout the day.	During the day (all day) on weekdays, and in the evenings or the weekend (one of both), patients can	During the day (all day) on weekdays, in the evenings and on weekends, patients can participate in an activity programme.

(e.g., work, training, sports and/or art), tailored to the patient's capabilities.				participate in an activity programme.	
<u>11. Use ROM (Routine Outcome Monitoring)</u> The multidisciplinary team uses Routine Outcome Monitoring (ROM), such as HoNOS and HKT-R, and translates the outcomes into both individual treatment policies and team policies to improve care.	The multidisciplinary team does not use ROM.	The multidisciplinary team uses ROM without feedback to team members or individual treatment policy.	The multidisciplinary team uses ROM and translates it into the individual treatment policy or into the team policy.	The multidisciplinary team uses ROM and translates this into both the individual treatment policy and the team policy.	The multidisciplinary team uses ROM structurally when evaluating the team performance, which leads to team-level adjustments; it also uses ROM as a standard component for each ZAG.

Domain 3. (Prevention) coercive measures					
**Criterium**	**Score 1**	**Score 2**	**Score 3**	**Score 4**	**Score 5**
<u>12. Stepped care</u><br>At any time of the day, a healthcare professional can provide 1-on-1 support and 2-on-1 support can be offered within 24 hours (except at night).	It is not possible to provide 1-on-1 support at any given time of day.	1-on-1 support can be provided during the week.	1-on-1 support can be provided during the week and during the weekend.	1-on-1 support can be provided during the week, during the weekend and at night.	1-on-1 support can be provided during the week, during the weekend and at night. 2-on-1 support can be provided within 24 hours (except at night).
<u>13a. Care process and consultation ICU</u><br>The stay at the ICU takes place according to the following criteria:<br>- It is possible to provide 1-on-1 care for a patient apart from the group.<br>- A psychiatrist/coordinating practitioner has at least one face-to-face contact with the patient per day (7 days a week).<br>- An ICU stay has a maximum duration of (*) days and can be extended up to two times.<br>- A colleague from outside the ward will be asked to check in after (*) days.<br><br>** FPA = 3 days, FPK = 5 days, FPC/PPC = 7 days*	None of the set criteria are met.	One of the set criteria is met.	Two of the set criteria are met.	Three of the set criteria are met.	All four of the set criteria are met.
<u>13b. Care process and consultation ESR/seclusion room</u><br>The stay at the ESR/seclusion room takes place according to the following criteria:<br>- The psychiatrist has face-to-face contact with the patient at least once a day (7 days a week)<br>- The team thinks creatively and out-of-the-box to limit the stay at the ESR.	None or just one of the set criteria are/is met.	Two of the set criteria are met.	Three of the set criteria are met.	Four of the set criteria are met.	All five of the set criteria are met.

- A colleague from outside the ward will be asked to check in after one day.<br>- The stay at the ESR (seclusion) has a maximum duration of (*) day(s).<br>- A stay in the ESR is only approved after obtaining advice from practitioners within the institution.<br><br>** FPA = 1 day, FPK = 4 days, FPC= 7 Days, PPC = 14 days*					
<u>14a. Coercive measures evaluation: team</u><br>Each coercive measure* is demonstrably evaluated with the FHIC team. The results of these evaluations are demonstrably used for the adaptation/improvement of the institution's practices with regard to coercive measures.<br><br>** Seclusion, involuntary medication, commitment according to BOPZ, level 4: commitment outside regular hours.*	Coercive measures are evaluated by the team in 0%-25% of cases.	Coercive measures are evaluated by the team in 25%-75% of cases.	Coercive measures are evaluated by the team in more than 75% of cases. However, this does not lead to an improved policy.	Coercive measures are evaluated by the team in 100% of cases. The use of the outcomes is unclear.	Each coercive measure is demonstrably evaluated by the team. The results of these evaluations are demonstrably used for the adaptation/improvement of the FHIC's practices with regard to coercive measures.
<u>14b. Coercive measures evaluation: patient</u><br>After each coercive measure*, the patient is offered the opportunity to evaluate. The patient's cooperation (or lack thereof) is documented, as well as whether they want to involve their next of kin. The outcomes of these evaluations are demonstrably used for the adaptation/improvement of the FHIC's practices with regard to coercive measures and the patient's crisis map.<br><br>** Seclusion, involuntary medication, commitment according to BOPZ, level 4: commitment outside regular hours.*	In 0%-25% of cases, the patient is offered evaluation after each coercive measure.	In 25%-75% of cases, the patient is offered evaluation after each coercive measure.	In more than 75% of cases, the patient is offered evaluation after each coercive measure. However, this does not lead to an improved policy.	In 100% of cases, the patient is offered evaluation after each coercive measure. The use of the outcomes is undear.	In 100% of cases, evaluation is demonstrably offered to the patient after each coercive measure. The outcomes of these evaluations are demonstrably used for the adaptation/improvement of the FHIC's practices with regard to coercive measures.

<u>15. Feedback on coercive measures</u><br>Sufficient attention is paid to feedback on coercive measures. This is reflected in the following criteria:<br>- A registration system keeps track of all coercive measures (e.g., Argus or MITS).<br>- The figures and trends of coercive measures are explained at least once a quarter.<br>- The multidisciplinary team receives feedback on the figures and trends of coercive measures.<br>- The multidisciplinary team evaluates the figures and trends of coercive measures.<br>- The outcomes of these evaluations are demonstrably used for the adaptation/improvement of the FHIC's practices with regard to coercive measures.	None or just one of the set criteria are/is met.	Two of the set criteria are met.	Three of the set criteria are met.	Four of the set criteria are met.	All five of the set criteria are met.

Domain 4. Safe care					
**Criterium**	**Score 1**	**Score 2**	**Score 3**	**Score 4**	**Score 5**
<u>16a. Incident response: employee/team</u><br>The following criteria are met:<br>- The need to receive care after an incident depends on the impact on healthcare provider(s), patient(s) or the quality of care. The team manager makes an appropriate assessment, taking into account the employee's resilience/burden at that time.<br>- After an incident, the employee(s) will be adequately taken care of by the team.<br>- After an incident, the employee(s) will be adequately taken care of by the team leadership.<br>- Within 24 hours, external care relief is available (from the facility, e.g., an Emergency Response Team).	None of the set criteria are met.	One of the set criteria is met.	Two of the set criteria are met.	Three of the set criteria are met.	All four of the set criteria are met.
<u>16b. Patient incident response</u><br>After an incident, follow-up care should be provided to patients in the ward. These are incidents with a potential impact on patients.	Patients are not provided with follow-up care after an incident.	After an incident, in 25%-75% of cases, follow-up care is provided to patients in the ward.	After an incident, in more than 75% of cases, follow-up care is provided to patients in the ward.	After an incident, in 100% of cases, follow-up care is provided to patients in the ward.	After an incident, follow-up care is provided to patients in the FHIC. Patient experiences and feedback are demonstrably used to improve ward quality (e.g., the ward climate).
<u>17a. Incident evaluation: with the team</u><br>Any incident/sentinel event that has an impact on the care provider(s), patient(s), or the quality of care is demonstrably evaluated with the team and the institution. The outcomes of these evaluations are	There is no incident evaluation within the team	Incidents are evaluated by the team in 25%-75% of cases	Incidents are evaluated by the team in more than 75% of cases. However, this does not lead to an improved policy	Incidents are evaluated by the team in 100% of cases. The use of the outcomes is unclear	Every incident is demonstrably evaluated by the team. The outcomes of these evaluations are demonstrably used for the adaptation/improvement

demonstrably used for the adaptation/improvement of the FHIC's practices with regard to the work and living environment					of the FHIC's practices with regard to the work and living environment
<u>17b. Incident evaluation: with the patient</u><br>Any incident/sentinel event that has an impact on the care provider(s), patient(s), or the quality of care is demonstrably evaluated with the patient. The outcomes of these evaluations are demonstrably used for the adaptation/improvement of the FHIC's practices with regard to the relevant patient's living environment and the crisis card.	There is no evaluation with the patient after an incident/sentinel event.	Incidents are evaluated with the patient in 25%-75% of cases.	Incidents are evaluated with the patient in more than 75% of cases. However, this does not lead to an improved policy.	Incidents are evaluated with the patient in 100% of cases. The use of the outcomes is unclear	Every incident is demonstrably evaluated with the patient. The outcomes of these evaluations are demonstrably used for the adaptation/improvement of the FHIC's practices with regard to the relevant patient's living environment and crisis card
<u>18a. Risk assessment – short term</u><br>The following criteria are met:<br>- Systematic monitoring takes place from the start to the end of a patient's admission, using observation instruments.<br>- Daily and periodic crisis monitoring take place through one or more instruments (e.g., BVC, the Kennedy Axis V, SDAS).<br>- The scores are used in the team analysis of a serious incident.<br>- The scores are used in the decision to scale up/down the intensive care.<br>- The scores and their application are anchored in methodology.	None or just one of the set criteria are/is met.	Two of the set criteria are met.	Three of the set criteria are met.	Four of the set criteria are met.	All five of the set criteria are met.
<u>18b. Risk assessment - long-term</u><br>If not present, a risk assessment tool will be used to determine the patient's risk factors and protective factors (e.g., HKT30-R, HCR-20 V3 and SAFROF)	For none of the patients, a long-term risk assessment is available at discharge	In less than 25% of patients, a long-term risk assessment is available at discharge	In 25%-49% of patients, a long-term risk assessment is available at discharge	In 50%-74% of patients, a long-term risk assessment is available at discharge	In 75%-100% of patients, a long-term risk assessment is available at discharge

Domain 5. Team structure						
**Criterium**		**Score 1**	**Score 2**	**Score 3**	**Score 4**	**Score 5**
19a. Small sociotherapeutic caseload: day shift<br>The available optimal basic staffing is:<br>4 sociotherapists for every 10 beds<br>(0.4 sociotherapist per bed)		An average of 2.0 or less sociotherapists for every 10 beds	On average 2.01 to 2.66 sociotherapists for every 10 beds	On average 2.67 to 3.33 sociotherapists for every 10 beds	On average 3.34 to 3.99 sociotherapists for every 10 beds	An average of 4.0 or more sociotherapists for every 10 beds
19b. Small sociotherapeutic caseload: evening shift<br>The available optimal basic staffing is: 4 sociotherapists for every 10 beds<br>(0.4 sociotherapist per bed)		An average of 2.0 or less sociotherapists for every 10 beds	On average 2.01 to 2.66 sociotherapists for every 10 beds	On average 2.67 to 3.33 sociotherapists for every 10 beds	On average 3.34 to 3.99 sociotherapists for every 10 beds	An average of 4.0 or more sociotherapists for every 10 beds
19c. Small sociotherapeutic caseload: night shift		*Note! See item 19C. to the application context: *FPA/FPK or **FPC/PPC*				
FPA, FPK *	The available optimal basic staffing is: 2 sociotherapists for every 10 beds	An average of 1.0 or less sociotherapists for every 10 beds	On average between 1.01 to 1.33 sociotherapists for every 10 beds	On average between 1.34 to 1.66 sociotherapists for every 10 beds	On average between 1.67 to 1.99 sociotherapists for every 10 beds	An average of 2 or more sociotherapists for every 10 beds
FPC, PPC **	Night shift at the ward starts at 20:00h. The ward is staffed by a healthcare professional (nurse/sociotherapist/other discipline with a healthcare background)	It is not possible to have a night shift employee (healthcare professional) in the ward immediately (within 15 minutes maximum).	A night shift employee (healthcare professional) can be present at the ward immediately (maximum within 15 minutes). This night shift employee can stay for up to 30 minutes.	A night shift employee (healthcare professional) can be present at the ward immediately (maximum within 15 minutes). This night shift employee can stay for up to 2 hours.	A night shift employee (healthcare professional) can be present at the ward immediately (maximum within 15 minutes). This night shift employee can stay for up to 4 hours.	A night shift employee (healthcare professional) can be present at the ward immediately (maximum within 15 minutes). This night shift employee can remain at the ward for the rest of the night.
20. Nurse/social worker (SPH)<br>At least 70% of the occupational group are nurses or social workers with higher education or academic graduates in social sciences (e.g., psychology, pedagogy)		Less than 40% of nurses/SPH have a higher education level or are academically trained in social sciences	40%- 49% of nurses/SPH have a higher education level or are academically trained in social sciences	50%- 59% of nurses/SPH have a higher education level or are academically trained in social sciences	60%- 69% of nurses/SPH have a higher education level or are academically trained in social sciences	At least 70% of nurses/SPH have a higher education level or are academically trained in social sciences
21a. (Coordinating) practitioner: Psychiatrist<br>There is 0.5 FTE psychiatrist position permanently available for direct patient care		There is 0-0.05 FTE psychiatrist available for every 10 beds	Between 0.05-0.2 FTE psychiatrist is available for every 10 beds	Between 0.2-0.35 FTE psychiatrist is available for every 10 beds	Between 0.35-0.5 FTE psychiatrist is available for every 10 beds	There is 0.5 FTE psychiatrist available for every 10 beds

for every 10 beds; this includes policy tasks such as attendance at meetings.<br>A resident or specialist in training of at least 0.5 FTE may be added as 0.1 FTE to the psychiatrist position.					
<u>21b. (Coordinating) practitioner: Diversity in disciplines</u><br>In addition to the psychiatrist, the team has two additional discipline coordinating practitioners (mental health psychologist, clinical psychologist, psychotherapist, nursing specialist for mental health). Optional disciplines are available/can be called in.	In addition to the psychiatrist, the team does not have a (coordinating) practitioner for another discipline. It is not possible to call in at least one of the above disciplines.	In addition to the psychiatrist, the team does not have a (coordinating) practitioner for another discipline. It is possible to call in at least one of the above disciplines.	In addition to the psychiatrist, the team has a (coordinating) practitioner for another discipline. It is not possible to call in at least one of the above disciplines.	In addition to the psychiatrist, the team has a (coordinating) practitioner for another discipline. It is possible to call in at least one of the above disciplines.	In addition to the psychiatrist, the team has two (coordinating) practitioners for another discipline.
<u>21c. (Coordinating) practitioner: FTE</u><br>In addition to the psychiatrist, there is 0.5 FTE coordinating practitioner present at the direct patient care unit for every 10 beds; this includes policy-related tasks such as attendance at meetings. A coordinating practitioner in training of at least 0.5 FTE may be added as 0.1 FTE to the FTE position of the coordinating practitioner.	There is 0-0.24 FTE coordinating practitioner available for every 10 beds	Between 0.25-0.49 FTE coordinating practitioner is available for every 10 beds	Between 0.5 and 0.74 FTE coordinating practitioner is available for every 10 beds	Between 0.75-0.99 FTE coordinating practitioner is available for every 10 beds	There is 1 FTE coordinating practitioner available for every 10 beds
<u>22. (Family) peer provider</u><br>Within the team, there is 1 FTE (family) peer provider available for every 10 beds	The team does not employ a (family) peer provider and does not actively use an peer provider within the organisation.	The team actively uses a (family) peer provider within its own organisation. The peer provider is not a fixed part of the team.	A (family) peer provider is employed for 0.1 - 0.5 FTE for every 10 beds	A (family) peer provider is employed for 0.51-0.99 FTE for every 10 beds	A (family) peer provider is employed for 1 FTE or more for every 10 beds
<u>23. Activity supervisor/ movement therapist/occupational worker</u><br>An activity supervisor/ movement therapist/occupational worker:	Does not meet any of the criteria; there is no activity supervisor/ movement	Meets one of the criteria set	Meets two of the criteria set	Meets three of the criteria set	Meets all four of the criteria set

- Is available to patients.<br>- Can offer activities in the ward where necessary.<br>- Actively participate in the multidisciplinary consultation.<br>- Is part of the team.	therapist/occupational worker				
<u>24. Additional disciplines</u><br>The following disciplines are available to the patient on indication:<br>- Social work/case manager<br>- Spiritual carer<br>- Legal consultant/confidential advisor<br>- Somatic specialist<br>- PMT/creative therapist<br>- Addiction physician<br>- System therapist<br>- Social mental health nurse (SPV)	None or one discipline is available for all patients	Two or three disciplines are available for all patients	Four or five disciplines are available for all patients	Six or seven disciplines are available for all patients	All eight disciplines are available for all patients
<u>25a. Team relationship: experience</u><br>Employees of the multidisciplinary FHIC team have at least two years of experience with crisis care for patients in forensic psychiatry, acute psychiatry, addiction care, ambulatory forensic care or elsewhere	0%-19% of employees have at least two years of experience in crisis care for patients, as described	20%-39% of employees have at least two years of experience in crisis care for patients, as described	40%-59% of employees have at least two years of experience in crisis care for patients, as described	60%-79% of employees have at least two years of experience in crisis care for patients, as described	80%-100% of employees have at least two years of experience in crisis care for patients, as described
<u>25b. Team relationship: mixed</u><br>FHIC's multidisciplinary team is mixed in terms of:<br>- Gender<br>- Age<br>- Cultural background<br>- Work experience (content)	The multidisciplinary team is not mixed on any of the elements	The multidisciplinary team is mixed on one element	The multidisciplinary team is mixed on two elements	The multidisciplinary team is mixed on three elements	The multidisciplinary team is mixed on all four elements
<u>26. Staffing</u><br>The multidisciplinary team is fully staffed, i.e., a minimum of unfilled vacancies**.** This applies to all disciplines. Flex workers do not count towards filling vacancies.	**The team has had a staffing rate of less than 50% in the last 12 months**	**The team has had a staffing rate of 50%-64% in the past 12 months**	**The team has had a staffing rate of 65%-79% in the past 12 months**	**The team has had a staffing rate of 80%-94% in the past 12 months**	The team has had a staffing rate of 95%-64% in the past 12 months

Domain 6. Culture/work method					
**Criterium**	**Score 1**	**Score 2**	**Score 3**	**Score 4**	**Score 5**
27. Vision<br>The team has a clearly described vision, derived from the FHIC model. All team members work according to this vision and communicate about it sufficiently with internal and external partners.	The team is not demonstrably working according to a vision derived from the FHIC model.	The team is demonstrably working according to a vision derived from the FHIC model, but the vision is not (clearly) described.	The team is demonstrably working according to a vision derived from the FHIC model and the vision is (clearly) described. However, team members do **not communicate sufficiently** about this with internal **and** external partners.	The team is demonstrably working according to a vision derived from the FHIC model and the vision is (clearly) described. However, team members do **not communicate sufficiently** about this with internal **or** external partners (either).	The team is demonstrably working according to a vision derived from the FHIC model and the vision is (clearly) described. Team members communicate **sufficiently about this with** internal **and** external partners.
28. Hospitality<br>The team's working methods align to the 'first five minutes methodology'. This is reflected in the following criteria:<br>- The checklist for the first five minutes of admission is followed (good preparation of the admission, good reception, introduction and arrangements).<br>- When assigning a mentor/personal supervisor to patients, the patient's preferences are taken into account as much as possible.<br>- At the start and end of the shift, team members take the initiative to contact patients.<br>- The team is not afraid to go beyond existing protocols if the situation so requires.	None of the set criteria are met	One of the set criteria is met	Two of the set criteria are met	Three of the set criteria are met	All four of the set criteria are met

<u>29. Presence</u><br>Professionals are present and available at the communal group at all times of the day. This is reflected in the following points:<br>- During the day, there is (are) always (a) sociotherapist(s) available at the communal group.<br>- There is no nursing office or it is left unstaffed as much as possible.<br>- The team discusses staffing arrangements during shift changes or consultation meetings.<br>- A (coordinating) practitioner is present and available at the communal group for at least one hour per day.	None of the set criteria are met	One of the set criteria is met	Two of the set criteria are met	Three of the set criteria are met	All four of the set criteria are met
<u>30. Attitude/treatment</u><br>- The team is constantly trying to establish contact, support, growth, and as little restraint as possible.<br>- The treatment of a patient does not vary per individual team member.<br>- The team uses insights from the patient's risk management plan to determine the patient's treatment.<br>- The team is aware of the patient's personal recovery process.<br>- The team will discuss treatment issues and has an introspective capacity.	None or just one of the set criteria are/is met	Two of the set criteria are met	Three of the set criteria are met	Four of the set criteria are met	All five of the set criteria are met
<u>31. Repression prevention</u><br>- The ward has as few house rules as possible (written and unwritten) – these are not institutional rules.<br>- The house rules are inventoried and evaluated for proportionality,	None of the set criteria are met	One of the set criteria is met	Two of the set criteria are met	Three of the set criteria are met	All four of the set criteria are met

subsidiarity and efficiency at least twice a year with the multidisciplinary team.<br>- Undesirable behaviour (e.g., drug use or aggression) is met with a consequence, without being penalized.<br>- After undesirable behaviour has been followed up by a consequence, the situation is discussed with the patient and the patient is given back the trust.					
<u>32. Family interventions</u><br>- Family members/next of kin are welcomed in a warm atmosphere.<br>- Family members/next of kin are actively involved in the patient's care.<br>- Forms of psycho-education are available for family members/next of kin.<br>- The ward offers the opportunity to join the patients for meals.<br>- The ward offers options for staying overnight in the ward or nearby (on the site is sufficient).	None or just one of the set criteria are/is met	Two of the set criteria are met	Three of the set criteria are met	Four of the set criteria are met	All five of the set criteria are met

Domain 7. Team development and dynamics					
**Criterium**	**Score 1**	**Score 2**	**Score 3**	**Score 4**	**Score 5**
33. Reflection on own actions<br>- Team members can look critically at their own actions and can handle feedback from others to improve their actions.<br>- The team members reflect with each other on their actions during consultation sessions, case meetings and daily care moments.<br>- Where necessary, team members consciously use their own (personal) experiences in their professional framework.<br>- Each team member participates in at least 6x2 hours a year of peer review or (group) supervision (such as moral deliberation) under the guidance of a supervisor (someone outside the team).<br>- Themes discussed during the peer review are tailored to the needs of the team.	None or just one of the set criteria are/is met	Two of the set criteria are met	Three of the set criteria are met	Four of the set criteria are met	All five of the set criteria are met
34a. Education and development: individual<br>At the FHIC, sufficient attention is paid to the development of individual employees. This is reflected in the following criteria:<br>- Team members can see perspective in their development.<br>- The team leader plays an observing role in the development of individual employees.<br>- All employees have a personal development/training plan.	At the FHIC, not enough attention is paid to the development of individual employees.	Attention is paid to the development of individual employees, but none of the criteria are met.	One of the set criteria is met	Two of the set criteria are met	All three of the set criteria are met

<u>34b. Education and development: team</u><br>All members of the multidisciplinary team receive annual training (minimum 4 parts of at least 3 hours) in the following areas:<br>- Addiction<br>- Psychopathology and psychopharmacology<br>- Suicide prevention and applicable guidelines<br>- Legal titles	The multidisciplinary team does not receive any annual training in the areas mentioned.	The multidisciplinary team receives annual training on one of the areas mentioned above.	The multidisciplinary team receives annual training on two of the areas mentioned above.	The multidisciplinary team receives annual training in three of the areas mentioned above.	The multidisciplinary team receives annual training on all four of the areas mentioned above.
<u>35. Conflict management and personal security</u><br>- A conflict management and personal security policy is available.<br>- The entire team knows and applies best practices for negotiation techniques, de-escalation techniques, and methods of physical safety that do not include pain stimuli.<br>- The use of the methodologies is evaluated on a half-yearly basis.<br>- The multidisciplinary team receives semi-annual physical resilience training.<br>- The multidisciplinary team receives de-escalation technique training every six months.	None or just one of the set criteria are/is met	Two of the set criteria are met	Three of the set criteria are met	Four of the set criteria are met	All five of the set criteria are met
<u>36. Team spirit</u><br>The team scores positive on the following items:<br>- The team experiences a sense of meaning at work: their work matters.<br>- The team pursues the same goals.<br>- The team agrees on the boundaries they set for the patients.<br>- The team has a collaborative structure.	The team does not score positively on any of these items.	The team scores positively on two points	The team scores positively on three points	The team scores positively on four points	The team scores positively on all five points

- The team has plenty of energy.					
37. Leadership<br>The team leaders (team managers and content managers) lead the team according to the following criteria:<br>- There is active leadership (leader is not afraid to intervene).<br>- Leadership applies the FHIC philosophy.<br>- The leadership is based on the five values of the FHIC (is responsive, attentive, competent, supportive and accountable).<br>- The leadership is the conservator of and inspiration for the FHIC culture.	The team leadership does not score positively on any of the criteria	The team leadership scores positively on one criterium	The team leadership scores positively on two criteria	The team leadership scores positively on three criteria	**The team leadership** scores positively on all four criteria
38. Collaborating leader<br>This is a role, not a job position. It may be filled by more than one person, and meets the following criteria:<br>- Provides 8 out of 36 hours direct assistance to patients as a team member.<br>- Actively monitors the ward's model compliance (working according to the FHIC workbook), actively implementing and securing the model.<br>- Monitors results from e.g., registration of coercive measures and safety notifications.<br>- Is present at least three times a week for the multidisciplinary consultation (digital whiteboard consultation).	None of the set criteria are met.	One of the set criteria is met.	Two of the set criteria are met.	Three of the set criteria are met.	All four of the set criteria are met.

Domain 8. Care organisation					
**Criterium**	**Score 1**	**Score 2**	**Score 3**	**Score 4**	**Score 5**
39. Admission and discharge<br>- There is a clear policy on the possible (treatment) goals that apply when a patient is eligible for admission to the FHIC.<br>- The referring party (internal and external) is aware of the applicable policy of admission and discharge at the FHIC.<br>- The team reaches clear agreements with the referring party about the admission.<br>- The team reaches clear agreements with the referring party about the patient being transferred.	None of the set criteria are met.	One of the set criteria is met.	Two of the set criteria are met.	Three of the set criteria are met.	All four of the set criteria are met.
40. Waiting list<br>It is possible to admit a patient within 24 hours at all times.	It is not possible to admit a patient within 24 hours.	It is possible to admit a patient within 24 hours in 0%-25% of cases.	It is possible to admit a patient within 24 hours in 25%- 49% of cases.	It is possible to admit a patient within 24 hours in 50%- 74% of cases.	It is possible to admit a patient within 24 hours in 75%-100% of cases.
41. Duration of the stay at FHIC<br>The stay at the FHIC has a maximum duration of * weeks and can be extended 2x if necessary after evaluation by the treatment team<br><br>* FPA = 3 weeks<br>FPK = 5 weeks<br>FPC = 7 weeks<br>PPC = not applicable	The standards for length of stay and extension are not followed for any of the patients	In 0%-25% of patients, the standards for length of stay and extension are followed	In 25%-49% of patients, the standards for length of stay and extension are followed	In 50%-74% of patients, the standards for length of stay and extension are followed	In 75%-100% of patients, the standards for length of stay and extension are followed
42. Knowledge of integrated care<br>The entire multidisciplinary team knows how integrated care works (all relevant internal and external parties that can refer to the	There is little knowledge within the team about how integrated care works.	-	Within the team, knowledge of how integrated care works is available; however, this	-	The team is fully aware of how integrated care works.

FHIC/where the patient is transferred after admission to the FHIC).			depends on the individual employee.		
<u>43a. Transition: consultation before admission</u><br>The consultation before admission meets the following criteria:<br>- Prior to admission, a consultation will take place with the referring party (internal or external).<br>- Prior to the admission, the emergence of the imminent crisis is analysed together with the referring party.<br>- It is possible to collaborate with the referring party during a number of moments prior to admission.	Consultation before admission at the referring party does not take place.	Consultation before admission at the referring party takes place but the criteria are not met.	One of the set criteria is met.	Two of the set criteria are met.	All three of the set criteria are met.
<u>43b. Transition: consultation during/after admission</u><br>During the admission to the FHIC, attention is paid to the crisis that occurred at the referring ward and to the post-admission transition. This meets the following criteria:<br>- The referring treatment team remains involved in the admission to the FHIC.<br>- The FHIC analyses the crisis at the referring party and advises on policy; the patient's agitation behaviour (aggression, self-mutilation, suicidal behaviour) and the interactions within the treatment teams are included in the crisis analysis and resolution.<br>- An admission report is available within two weeks of discharge<br>- Upon discharge, all patients have a crisis observation plan	None or just one of the set criteria are/is met	Two of the criteria are met	Three of the criteria are met	Four of the criteria are met	All five of the criteria are met

- Upon discharge, all patients have a risk management plan					

Domain 9. Spatial design					
**Criterium**	**Score 1**	**Score 2**	**Score 3**	**Score 4**	**Score 5**
<u>44. Ward: a healing environment</u><br>In the ward, structural attention is paid to:<br>- The physical environment is designed to promote the well-being of patients, next of kin and team members (sufficient daylight, fresh air, plants, use of natural materials).<br>- Attention is paid to a healthy living environment. The multidisciplinary team has a joint responsibility to keep the common (outdoor) areas clean and tidy.<br>- Patients are, based on their capacity, coached by the team to maintain their own environment and the common living environment in a healthy way.<br>- The ward uses an instrument (e.g., OAZIS) to test and improve the extent of the healing environment.	None of the set criteria are met.	One of the set criteria is met.	Two of the set criteria are met.	Three of the set criteria are met.	All four of the set criteria are met.
<u>45a. Ward: lockable rooms</u><br>Single rooms with a shower and toilet are available and can be locked by the patient	No	-	-	-	Yes
<u>45b. Ward: diversity in meeting areas</u><br>The ward has a variety of places where the patient can go.	No	-	-	-	Yes
<u>45c. Ward: open desk/workstations</u><br>The ward has an open desk or workstations	No	-	-	-	Yes
<u>45d. Ward: outdoor area/patio</u><br>The ward has an outdoor area/patio that is accessible to patients from the ward	No	-	-	-	Yes
<u>45e. Ward: comfort room</u><br>The ward has a comfort room that is accessible to patients without a key/without help.	No	-	-	-	Yes

<u>45f. Family room</u><br>Patients can use a family room located in or near the ward.	No	-	-	-	Yes
<u>46. The IC (Intensive Care)</u><br>The IC meets the following criteria:<br>- The IC is a structural unit equipped with a number of ICUs and ESRs<br>- This is a physical room integrated into the FHIC, where the team provides 1-on-1 supervision (continuity of care)<br>- There is a closed garden<br>- There is plenty of room for different purposes and activities<br>- The patient entry provides access to a consultation room on the IC	None or just one of the set criteria are/is met.	Two of the set criteria are met.	Three of the set criteria are met.	Four of the set criteria are met.	All five of the set criteria are met.
<u>47. ICU room (Intensive Care Unit)</u><br>The ICU meets the following criteria:<br>- These are individual rooms<br>- There is a sitting and sleeping area plus sanitary facilities<br>- If there are several ICU's, these will not be interconnected<br>- The patient controls the (day) light, temperature and media	None of the criteria are met	One of the criteria is met	Two of the criteria are met	Three of the criteria are met	All four of the criteria are met
<u>48. The Extra Secure Room (ESR)/seclusion</u><br>The ESR meets the following criteria:<br>- There is a window where the patient controls whether or not to see the healthcare provider<br>- There is a toilet with a sink<br>- A touch screen is provided to enable the patient to stay in touch with the team<br>- The patient controls the (day) light, temperature and media<br>- There is a hallway with sanitary facilities	**None or just one of the set criteria are/is met.**	**Two of the set criteria are met.**	**Three of the set criteria are met.**	**Four of the set criteria are met.**	All five of the set criteria are met.

Domain 10. Quality					
**Criterium**	**Score 1**	**Score 2**	**Score 3**	**Score 4**	**Score 5**
49. Innovation and improvement<br>Sufficient attention is paid to innovation and improvement. This is reflected in the following criteria:<br>- The atmosphere is highly innovative, the team is open to new ideas and thinks out of the box to continue improving.<br>- The team is supported by the organisation in the development of new ideas (employees are given room and time for this, project leaders/staff members are available to support this).<br>- The team and leaders regularly monitor the quality of care; for example, they regularly review the FHIC Monitor with the team. These results are used to improve the patient care and support. For example, the PDCA cycle is completed for support.<br>- There is a structural exchange of knowledge and experience between internal and external (outside the organisation) teams.<br>- National developments related to FHIC and new interventions are actively monitored by the team and shared within the team and the organisation.	None or just one of the set criteria are/is met.	Two of the set criteria are met.	Three of the set criteria are met.	Four of the set criteria are met.	All five of the set criteria are met.
50a. Quality improvement: work environment<br>Sufficient attention is paid to monitoring the working environment in the ward. This is reflected in the following criteria:	None of the set criteria are met.	One of the set criteria is met.	Two of the set criteria are met.	Three of the set criteria are met.	All four of the set criteria are met.

- The work environment is measured at least every six months using an instrument (e.g., the LGWCI) or an employee satisfaction survey.<br>- When monitoring the work environment, attention is paid to leadership, team performance, job motivation and employer/employee obligations.<br>- The results are demonstrably linked back to the team.<br>- The results are demonstrably used to improve the quality of the work environment.					
<u>50b. Quality improvement: social climate</u><br>Sufficient attention is paid to monitoring the social environment in the ward. This is reflected in the following criteria:<br>- The social environment is measured at least every six months among employees and patients (e.g., through the GCI/GCI-R or EssenCES)<br>- The results are demonstrably linked back to the patients.<br>- The results are demonstrably linked back to the professionals.<br>- The results are demonstrably used to improve the quality of the ward.	None of the set criteria are met.	One of the set criteria is met.	Two of the set criteria are met.	Three of the set criteria are met.	All four of the set criteria are met.
